# A Robot-Assisted Surgical System Using a Force-Image Control Method for Pedicle Screw Insertion

**DOI:** 10.1371/journal.pone.0086346

**Published:** 2014-01-22

**Authors:** Wei Tian, Xiaoguang Han, Bo Liu, Yajun Liu, Ying Hu, Xiao Han, Yunfeng Xu, Mingxing Fan, Haiyang Jin

**Affiliations:** 1 Department of Spine Surgery, Beijing Jishuitan Hospital, Beijing, China; 2 Medical Center, Tsinghua University, Beijing, China; 3 Shenzhen Institutes of Advanced Technology, Chinese Academy of Sciences, Shenzhen, China; The Ohio State University Medical Center, United States of America

## Abstract

**Objective:**

To introduce a robot-assisted surgical system for spinal posterior fixation that can automatically recognize the drilling state and stop potential cortical penetration with force and image information and to further evaluate the accuracy and safety of the robot for sheep vertebra pedicle screw placement.

**Methods:**

The Robotic Spinal Surgery System (RSSS) was composed of an optical tracking system, a navigation and planning system, and a surgical robot equipped with a 6-DOF force/torque sensor. The robot used the image message and force signals to sense the different operation states and to prevent potential cortical penetration in the pedicle screw insertion operation. To evaluate the accuracy and safety of the RSSS, 32 screw insertions were conducted. Furthermore, six trajectories were deliberately planned incorrectly to explore whether the robot could recognize the different drilling states and immediately prevent cortical penetration.

**Results:**

All 32 pedicle screws were placed in the pedicle without any broken pedicle walls. Compared with the preoperative planning, the average deviations of the entry points in the axial and sagittal views were 0.50±0.33 and 0.65±0.40 mm, and the average deviations of the angles in the axial and sagittal views were 1.9±0.82° and 1.48±1.2°. The robot successfully recognized the different drilling states and prevented potential cortical penetration. In the deliberately incorrectly planned trajectory experiments, the robot successfully prevented the cortical penetration.

**Conclusion:**

These results verified the RSSS’s accuracy and safety, which supported its potential use for the spinal surgery.

## Introduction

Pedicle screw fixation plays an important role in many spinal surgeries, providing superior post-operative spinal stability. As the morphology of the pedicle is complex and due to its proximity to a number of significant tissues (e.g., spinal cord and nerve root), screw misplacement might lead not only to a decreased stability but also to neurological, vascular, and visceral injuries [1], [2]. In practice, the incidence of inappropriate screw insertion is up to 10 percent, and half of these insertions result in critical injuries to the patient [3], [4]. New techniques to improve the pedicle screw placement are thus required.

Due to the higher requirements of spinal surgery, robots are ideal candidates for surgical assistants, as they can achieve superior levels of precision, are not affected by fatigue and can perform repetitive tasks without decreased performance [5], [6]. In recent years, a variety of robots have been introduced for spinal surgical applications. One representative instance is the SpineAssist (Mazor Surgical Technologies [HQ] Ltd., Cesarea, Israel), which has been the only commercial robot used in spine surgeries [7]–[10]. This surgical robot was designed with a steward parallel robot and a guiding rod fixed on the moving platform. During the operation, the robot fixed on the patient can adjust the position/orientation of the guiding rod, through which the surgeon can manually drill the screw paths. Recently, Mazor Robotics introduced the Renaissance, a new version of the SpineAssist, which, despite retaining the SpineAssist’s core technologies, had a complete overhaul of the software and user interface. The Renaissance also has new features such as the C-OnSite, which permits the acquisition of 3D images using a normal C-arm [6]. These systems can effectively overcome the physiological hand tremor of the surgeon so that the operation stability can be improved. Their primary innovation was the reduced size and weight, which permitted its direct attachment to the patient’s body structure. This ability can simplify the registration on pre- and intra-operative images because neither tracking nor immobilization are needed, as no relative motion between the patient and the robot is possible [6]. However, the reduced working volume of Renaissance robots also makes them less able to withstand reactive forces, which in the case of drilling, can reach 15 N [6], [8]. Furthermore, as the pedicle drilling operation is still performed manually by the surgeon, the operation precision and the operation state recognition still depend on the surgeon’s experience. Findings from the aforementioned studies were encouraging and suggested that robot-assisted surgery could be used to increase the accuracy of pedicle screw placement in spinal surgery [6]–[10]. Further research, however, is clearly needed to confirm the applicability of robot-assisted approaches.

Most current robots are primarily fast and robust positioning machines, which guarantee precision by means of mechanical stiffness and visual information alone [6]. In addition to the vision information, the force feedback is considered to be a useful sensing signal during the operation [11]. Some researchers have tried to equip the spine robots with force information analyzers. Wang et al. proposed a milling robot for cutting operations in spinal surgery to detect the milling depth and to judge whether the cutting state is normal by recording the entire force in each layer [12]. However, this process was not a real-time state recognition. Lee et al. developed an instrument for sensing the thrust force along with the torque. By processing these signals, the breakthrough status in the drilling could be detected, and the drilling motion could be constrained in real time [13]. However, the drilling feed rate was too fast (26 mm/s) to meet the safety requirements of clinical surgery. We have also previously proposed a novel spinal surgical system, the Robotic Spinal Surgery System (RSSS), which was designed to fuse image information and thrust force signals to sense the operational state in the pedicle screw insertion operation and to prevent the potential penetration [14].

However, the validation and safety of the RSSS, which are most important for an operational robot, have not been tested. Therefore, in this article, we reported the technique of pedicle screw placement using the RSSS and evaluated the accuracy and safety of this technique in sheep vertebra experiments.

## Materials and Methods

### RSSS System

**Figure 1:**
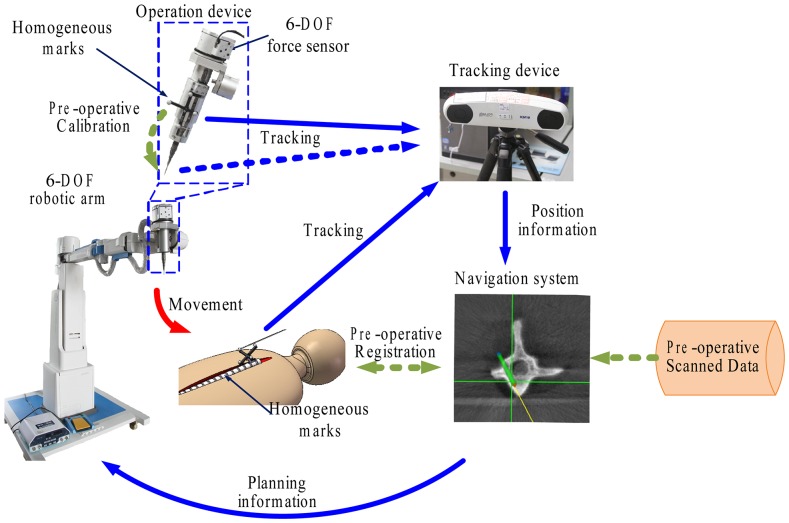
Architecture of the robotic spine-assisted surgery system.

The details of the Robotic Spinal Surgical System (RSSS) and the associated surgical tool have been previously described [14]. Briefly, the RSSS consisted of optical tracking, navigation, and planning systems and a surgical robot equipped with a 6-DOF force/torque sensor. Each part of the system was designed with the workspace and necessities for operation in mind (Fig. 1).

### Navigation and Planning System

The 3D images composed of the sagittal, coronal, and axial planes created by the ARCADIS Orbic 3D (Siemens Medical Solution, Erlangen, German) were transferred to the navigation system. These images were then matched with the real patient’s anatomy by using the optical tracking system and were used for precise trajectory planning. After designing the surgical path, this information was transferred to the robot.

Intra-operatively, the navigation system was also able to integrate the tracking information into the 3D image coordination, which was displayed on-screen in real time. Furthermore, the admission path on the spine and the real-time posture of the instruments were also shown on the screen.

### Tracking System

The infrared optical tracking system consisted of a camera (NDI brand: Polaris), homogeneous markers, and a pointer. The markers were attached to the robot and the vertebra to be navigated. The pointer was used to define the point coordinates in space. The tracker monitored the positions and movements of the robot end-effector as well as the surgical area by tracking these marks.

The registration and calibration must be performed prior to the operation. We obtain the positions of the selected fiducial points on the image displayed in the planning system and measure the corresponding position of these points by using the optical tracking system in the real-world coordinates. Thorough repeated selections and measurements, the coordinate frame of the image and that of real patient’s body were synchronized [15], [16]. Four infrared markers are fixed on the operation tool of the RSSS and are tracked by the tracking device. The positions and orientations of the infrared markers are converted to the operation tool’s position and orientation after calibration [17].

The Model Predict Control (MPC) was used to perform compensation strategies. Intra-operatively, the movement of the surgical target is tracked in real time, and the period and amplitude are predicted. The predicted movement is used as the compensation input for the motion control of the robot [18].

### Robotic System

The robot system was composed of a 6-DOF robotic arm, which was used for accurate positioning, and a 1-DOF operation tool used for the drilling screw path. The end of the robot arm was equipped with a 6-DOF force/torque sensor to track the generalized force acting on the drilling device during the drilling process. In the actual operation, the feed speed was 0.5 mm/s, and the drilling speed was 12,000 rpm. The force signal was sampled by the force sensor and was transmitted into a data acquisition card (DAQmx card) with a sampling rate of 1 KHz. These data were collected into a buffer with a length of 50 and were sent to a dynamic link library (DLL) with a frequency of 20 Hz. The force signals were collected and analyzed.

### State Recognition Algorithm

**Figure 2:**
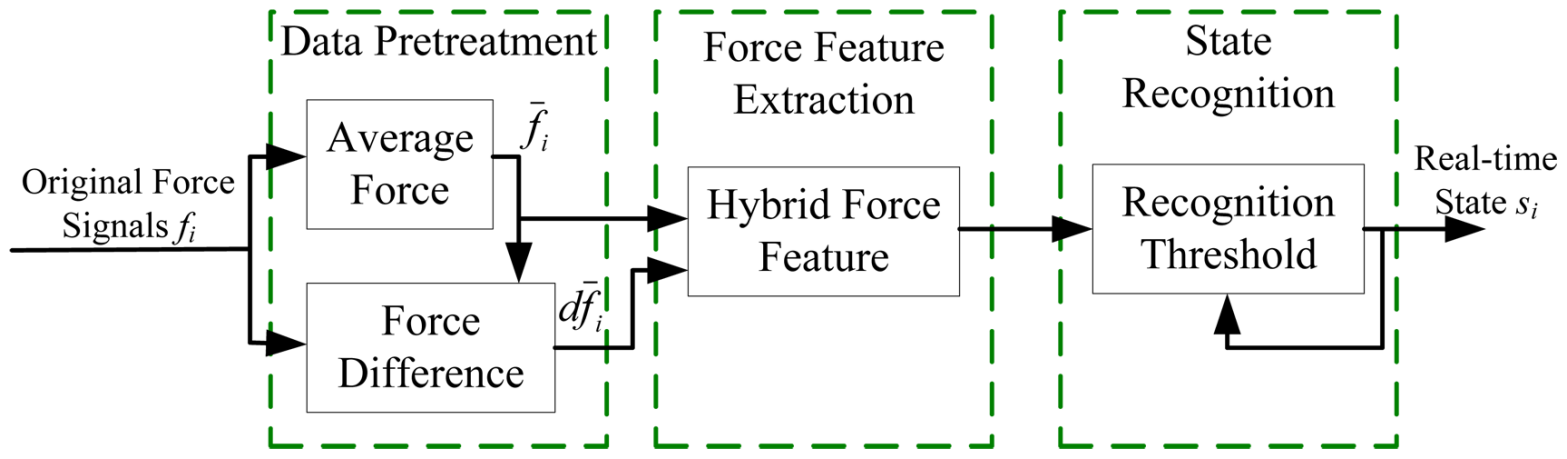
State recognition algorithm.

As shown in Figure 2, the algorithm is consisted of three main parts: data pretreatment, force feature extraction and state recognition. The original force signals 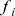 (*i* = 1,2,….,*n*) are pretreated, and their short time average value 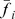 and the force difference 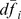 are calculated as the force features. In the force feature extraction module, the hybrid force features, which combine the average value and force difference, are obtained, and the state recognition model is then presented. The hybrid force features and the feature threshold are compared, and the current state can then be judged according to the force signals.

The force features in cortical tissue and cancellous tissue present different characteristics. After penetrating the first cortical layer, the force signal of the cortical layer is recorded and used to calculate the feature threshold of the second cortical bone. As continued drilling after the second cortical penetration will cause potential injuries, when the feature threshold of the second cortical bone is detected, the drilling process is stopped.

**Figure 3:**
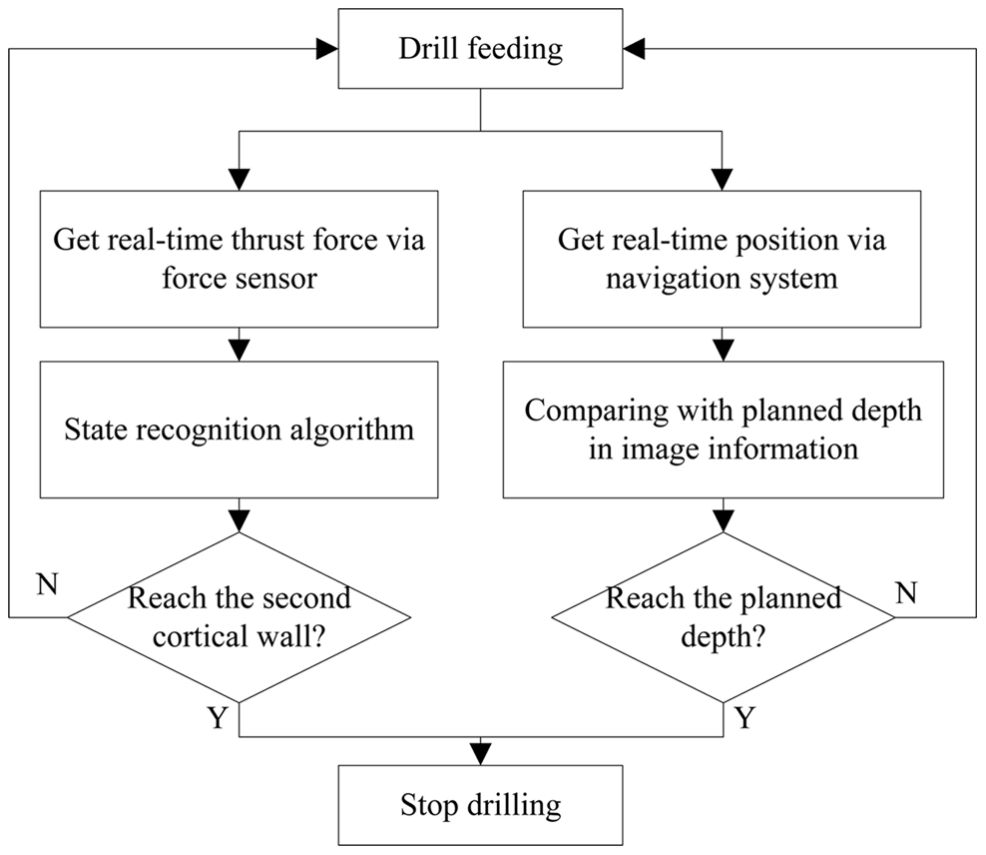
The control algorithm by the coordinated image and force information.

As shown in Figure 3, the RSSS used the image message and the force signals to sense the different operation states and to prevent potential cortical penetration in the pedicle screw insertion operation.

### Surgical Outflow

**Figure 4:**
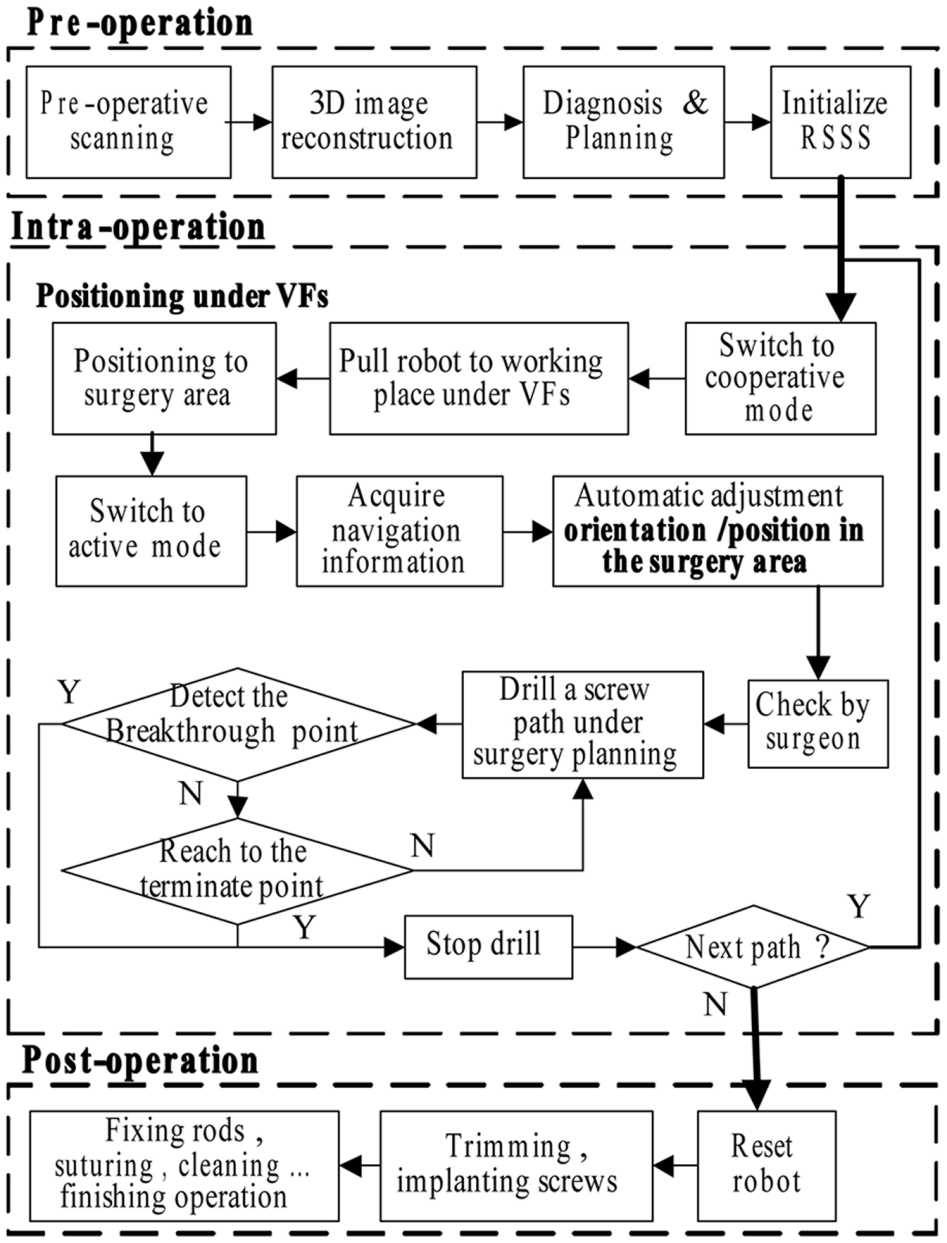
The procedure of screw insertion operation with the RSSS.

As shown in Fig. 4, the surgical workflow was mainly divided into two stages. In the pre-operation stage, the homogeneous marks were fixed onto the patient’s vertebrae and were scanned by the C-arm. The C-arm images were loaded to reconstruct the optimal position and dimensions of the trajectory plans. The RSSS was then initialized, and the pedicle drilling device was calibrated in the system.

In the intra-operation stage, the surgeon dragged the drilling device and located it close to the entry position in the cooperative control mode. Switching to the automatic control mode, the robot accurately adjusted to the planned trajectory. After being checked by the surgeon, the robot began to drill into the vertebra. The navigation information and the thrust force signal were recorded on a real-time basis. The robot stopped drilling at a set end point or was alternatively guided by the force control. The robot immediately stopped drilling if the force signal was abnormal. The robot motion, drilling, and insertion were repeated for all the required implants. The surgeon then inserted the screws and rods, sutured and cleaned the wound, and completed the operation.

### Sheep Spine Experiment

**Figure 5:**
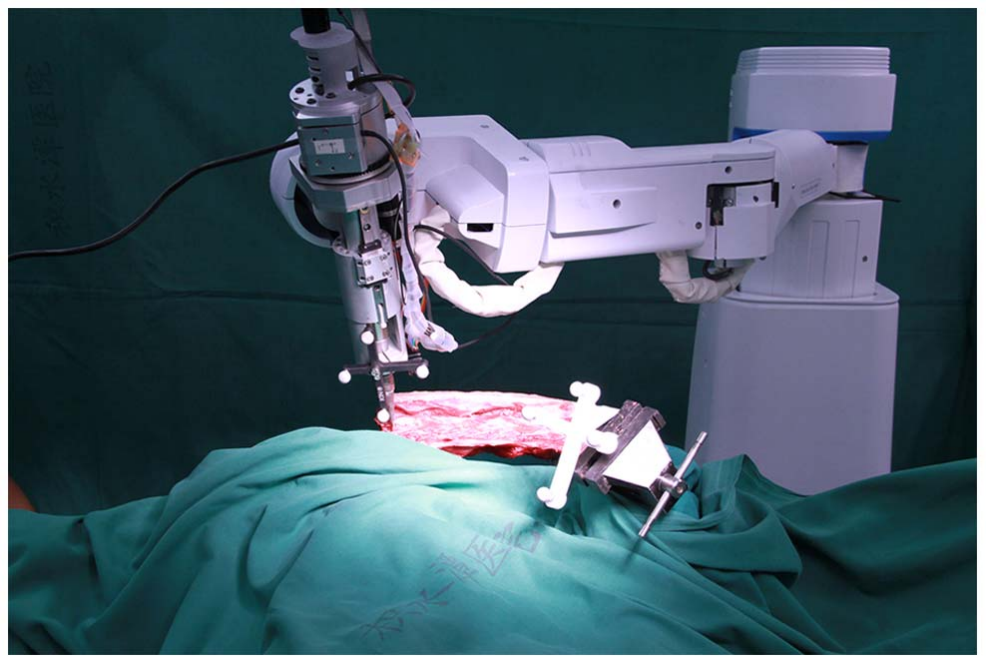
Experimental setup with a sheep spine.

All the specimens were provided and permitted for experimental use by a slaughterhouse (Beijing Huadu Meat Product Company). With the approval of the Institutional Animal Care and Use Committee of Peking University, a total of 8 sheep lumbar spines (2–3 years old, 60.1±7.2 (58–73) kg) were used in this accuracy evaluation. Two to five lumbar spinal levels from each sheep were randomly used, and a total of 32 levels were instrumented in this test. The 4.5-mm-diameter, 40-mm-long screws were used for the test. The sheep spine was immobilized on the operating table in the prone position, and the procedures (Fig. 5) were performed as the surgical flow described.

### Evaluation

**Figure 6:**
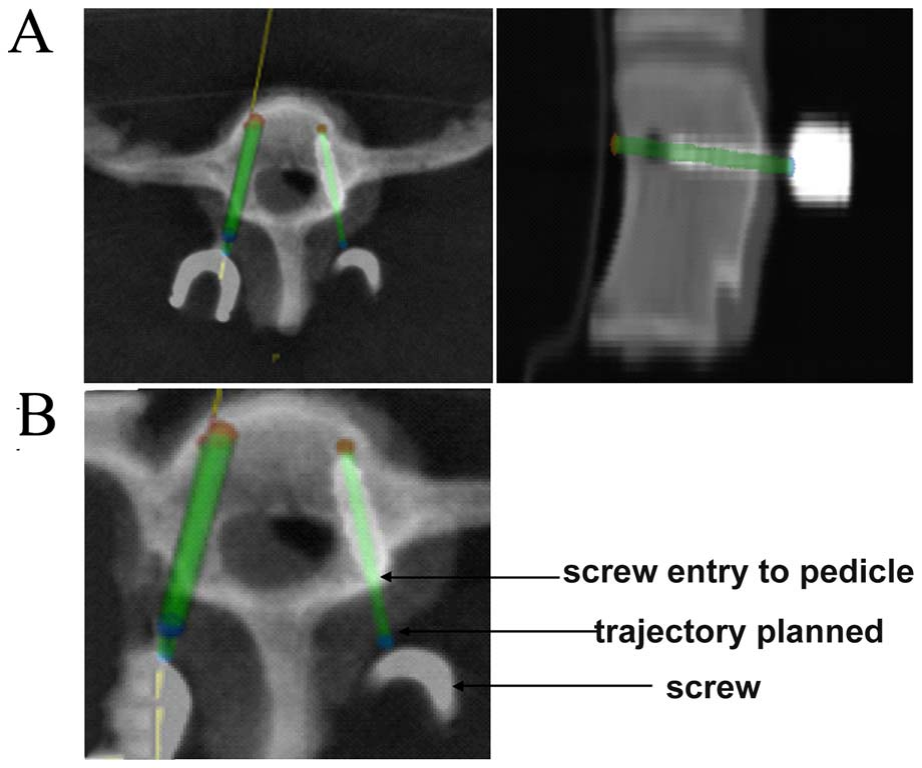
Postoperative computer tomography scans indicating the results of the sheep vertebrae study for the safety analysis.

After the operation, a postoperative CT scan was performed. A blind evaluation of the screw positions was performed by two radiologists. Any cortical breaches of the pedicle by the screw were measured in millimeters in the medial, lateral, cranial, or caudal directions, according to the Gertzhein and Robbins classification [8]. The discrepancies between the surgeon’s plan and the actual placements were also measured at the entry point and at the angles in the axial and lateral views [10], [15]. The relative tendency of the direction of the deviations was analyzed in each dimension (Fig. 6).

### Analysis of Thrust Force

Each force signal was sampled and analyzed to judge whether the robot automatically detected the different drilling state and prevented the potential cortical penetration. The computational details of the force signal and its algorithms were described in our previous report [14].

### Safety Test

For deliberate perforations, five vertebral levels were chosen from the aforementioned set of 8 vertebral bodies. Six trajectories were planned to perforate the inner canal or the outer cortex bone to explore whether the robot could detect the abnormality of the force signal and immediately stop drilling. The surgical procedures and the force signal analysis were performed as prescribed above.

## Results

**Figure 7:**
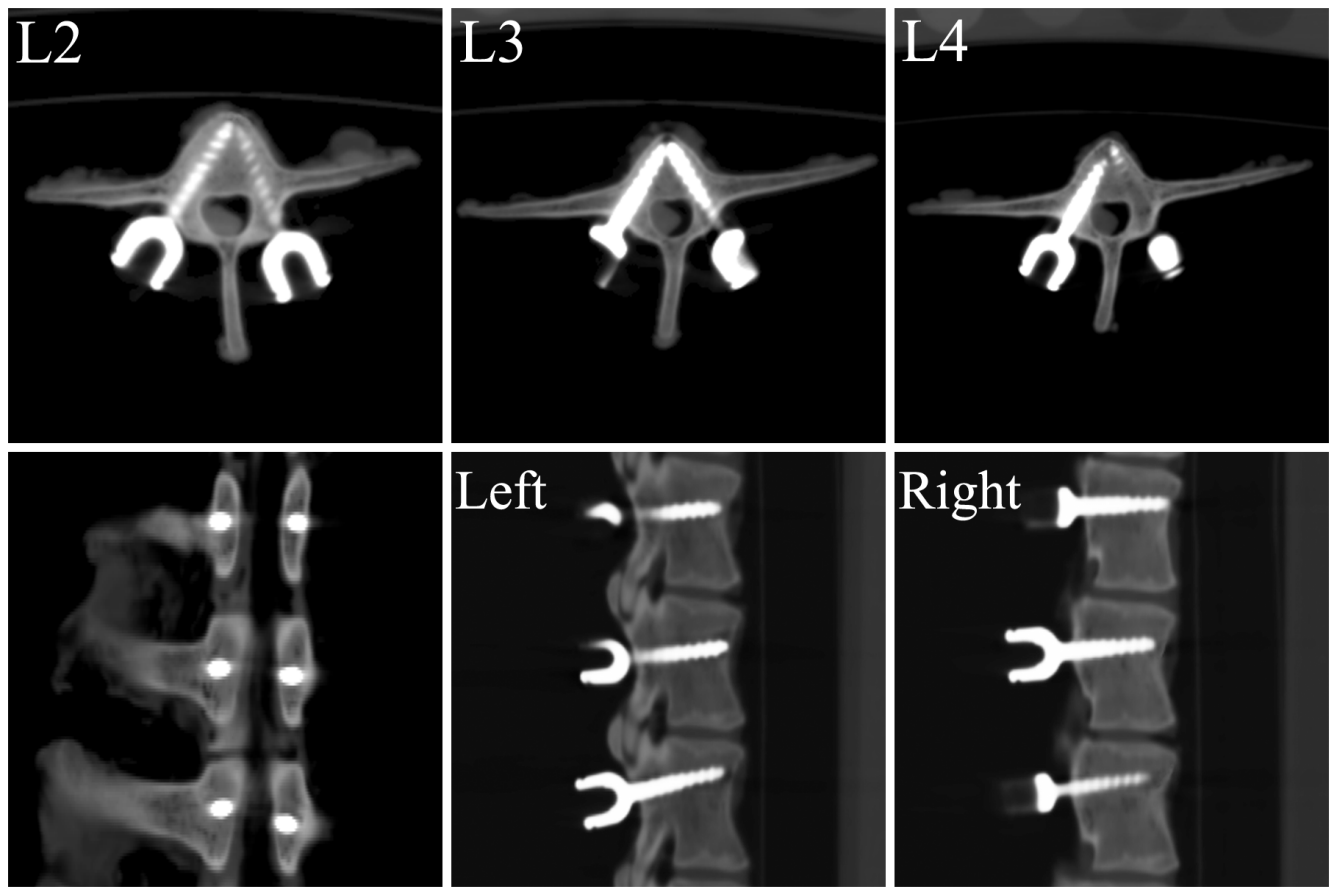
Screw position accuracy measurements as determined on postoperative CT scans. A, Planned merged over actual placement. B, Comparison between the panned and inserted screw at the entry point and angles.

For 32 of the pedicles, the surgical procedures were smoothly performed using the RSSS. According to the post-operation CT image data, the pedicle screw placements were sufficient because there was no perforation of the spinal canal or any unexpected malpositioning. According to the Gertzbein-Robbins classification, 32 screws fell into the group A, i.e., good screw positions (Fig. 7). Furthermore, as demonstrated in Table 1, there was a discrepancy between the planned and the actual placements at the entry points and the angles in the axial and lateral views. These results indicated that the average deviations of the entry points in the axial and sagittal views were 0.60±0.41 and 0.53±0.36 mm, and the average deviations of the angles in the axial and sagittal views were 2.08±2.31° and 2.40±2.09°.

**Table 1:** The accuracy and error analysis of the experiments.

Screw serial no.	Specimen no.	Level and side	Deviation of entry site (mm)		Deviation of angles (°)		Error (mm)	
			Lateral	Axial	Lateral	Axial	Calibration	Registration
1	A	L3L	0.8	0.2	1.2	1.0	0.16	0.15
2	A	L3R	0.3	0.3	6.9	0.2		
3	A	L4L	1.2	0.6	0.0	2.0		
4	A	L4R	0.6	1.2	1.3	0.5		
5	B	L4L	0.8	0.2	6.2	1.2	0.10	0.14
6	B	L4R	0.3	1.3	5.1	7.9		
7	B	L5L	0.2	0.4	3.8	1.0		
8	B	L5R	0.4	0.3	1.1	0.1		
9	C	L3L	0.3	0.7	2.3	0.4	0.11	0.20
10	C	L3R	1.0	1.2	3.1	0.0		
11	C	L4L	0.4	0.5	0.0	8.3		
12	C	L4R	0.0	0.3	0.0	1.1		
13	C	L5L	1.2	1.5	0.3	1.8		
14	C	L5R	0.5	0.6	3.1	8.4		
15	D	L4L	0.4	1.2	2.1	4.6	0.13	0.18
16	D	L4R	1.3	0.8	1.1	1.3		
17	D	L5L	0.4	0.4	0.4	1.5		
18	D	L5R	0.3	0.3	0.8	0.9		
19	E	L4L	0.4	1.1	3.4	2.4	0.10	0.11
20	E	L4R	0.3	1.2	2.7	0.5		
21	E	L5L	0.6	0.2	4.5	2.1		
22	E	L5R	0.0	0.4	3.8	1.2		
23	F	L3L	0.1	0.5	0.4	2.9	0.10	0.13
24	F	L3R	0.2	0.4	4.2	1.2		
25	F	L4L	0.4	0.3	0.2	3.2		
26	F	L4R	0.4	0.3	1.2	4.1		
27	G	L2L	0.8	0.6	1.1	1.5	0.14	0.21
28	G	L2R	0.4	1.2	0.2	2.9		
29	G	L3L	0.9	0.1	2.8	0.0		
30	G	L3R	0.5	0.5	2.1	2.1		
31	G	L4L	1.3	0.3	7.4	0.2		
32	G	L4R	0.4	0.1	4.1	0.1		
Average			0.53	0.60	2.40	2.08	0.12	0.16
SD			0.36	0.41	2.09	2.31	0.02	0.04

**Figure 8:**
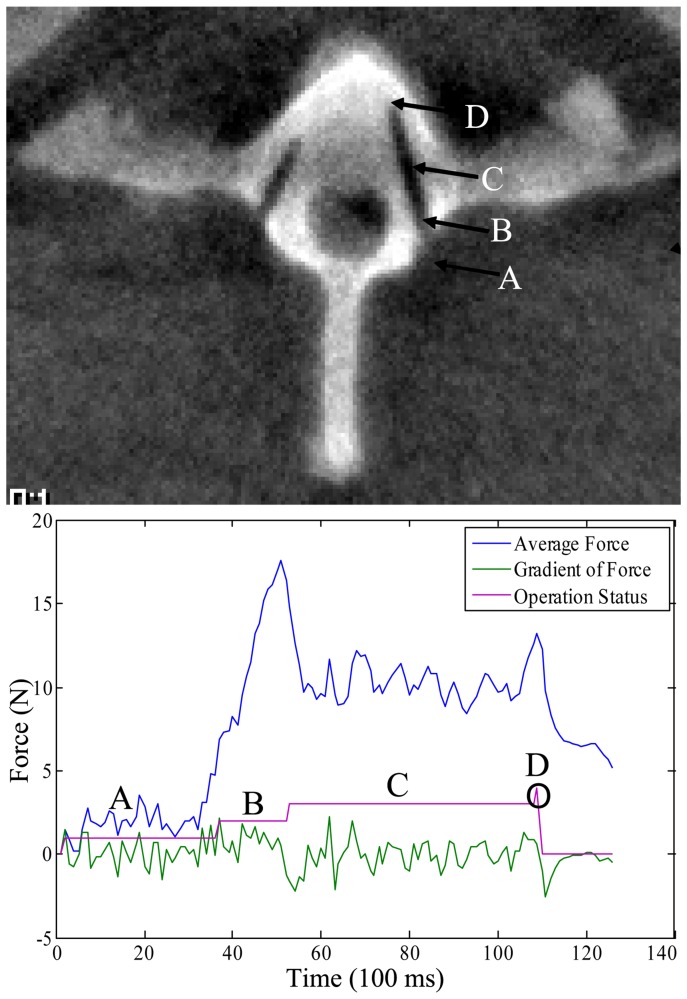
The representative force signal maps of the experiments. A, the initial state, (first cortical bone remains untouched); B, the first cortical state (an obvious peak); C, the cancellous state (a low force level) and D, the second cortical state (the force signal began to increase).

As demonstrated in Fig. 8, a typical curve was mainly divided into four states: the initial state (the drill did not touch the first cortical bone); the first cortical state (drilled the first cortical bone; an obvious peak); the cancellous state (drilled the cancellous tissue; a low force level) and the second cortical state (the drill approached the second cortical bone, and the force signal began to increase). This fourth stage was the sign for the potential second cortical penetration that with continued drilling could lead to potential neurological, vascular, or visceral injuries. Therefore, this state was set as the drilling end. In all 32 experiments, the force sensor successfully recognized this state and immediately stopped without any cortical penetration.

**Figure 9:**
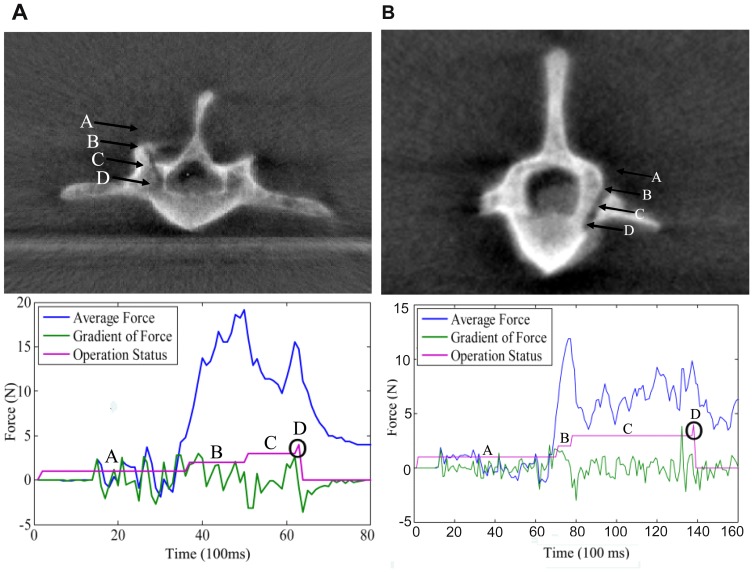
Deliberately incorrectly planned trajectory. When the drill touched the second cortical bone, there was an obvious peak in the force signal, and the robot spontaneously stopped drilling.

In the wrongly planned trajectory, when the drill reached the second cortical bone (inner or outer cortical bone), there was an obvious peak in the force signal, and the robot successfully stopped drilling (Fig. 9). All six wrongly planned trajectories did not penetrate the inner canal or the outer cortical bone.

## Discussion

In recent years, a variety of robots for spinal surgical applications have been introduced; these robots could be mainly divided into two types. One type is used for guidance of the manual operations, in which the robot gives the position and orientation of the screw path but the drilling process is still manually performed by the surgeon [6]. The other type of robotic system is regarded as a fully automatic operation system. The RSSS belongs to this latter type. In this case, the robotic system can automatically perform both positioning and pedicle screw path drilling according to the surgical plan under the supervisory of the surgeon. These automated surgical robotic systems have good positioning and maintenance properties [6], [19], In addition, the RSSS can recognize the drilling states by the real-time force sensing.

As safety remains the most important issue for the robotic system, it is therefore most important to improve the safety and stability of the robotic procedures. In the RSSS, we have fully prioritized the safety of the robotic systems [14]. As previously mentioned, the gravity effects of the robotic arm were balanced except for the first prismatic joint [14]. In case of accidents, such as the loss of power or over-speeding, the robotic arm should not move down to harm the patient. Furthermore, the robot could be controlled in two modes, the cooperative mode for a coarse position and the active mode for a precise position, thereby maximizing the flexibility of the end-effector and enhancing the accuracy and stability of these operations [14]. In addition, the mill was set to be constant at a low feeding speed (0.5 mm/s), which was not only more convenient for the system control but also provided a better safety [12] because the surgeon could anticipate where the mill would be next. Although the drilling could be automatically processed by the robot, the procedure remains under the supervision of the surgeon, and the surgeon could control the robot using a manual panel. An emergency stop function was provided in case of unexpected occasions. Most importantly, combined with the real-time version information, the real-time force sensing was used to recognize different drilling states. Therefore, this image-force fused information improved the precision and safety of the robot.

The information from the force signal and the image messages had advantages and disadvantages. For the image information, the 3D model was preoperatively prepared, which was very easy and intuitive. Once the drill position and orientation were designed, the drilling path was determined, and the position of the drill tip could be supervised. However, because of the radiation, the navigation image always had to be acquired pre-operatively and could not be acquired continuously during the operation. As the patient might move, there might be a poor congruence between the 3D images and the real object, which could decrease the precision and might even mislead the surgeon [20]. For the force signals, it was the real-time sensation of the operational signal, which could find the different drilling states and prevent the potential cortical breach [11], [14]. In some situations, such as when the drilling position was close to the edge of the bone or in the case of an osteoporotic bone, the features of the force signal was not very clear in that the cortical and cancellous tissues were hardly separated. Therefore, the two types of information were merged together to better recognize the state of drilling and to improve the safety of the operation. This merger was a distinct property compared to any other surgical robot system in clinical use.

To validate this system, 32 pedicle screw insertions were conducted in sheep spines. In each screw insertion, the full procedures from the preoperative to the postoperative tasks were tested and evaluated to determine whether these tasks were proper to be applied to the clinical fields. All the screws fell into the group A, i.e., good screw positions, and the average deviations of the entry points in the axial and sagittal views were 0.50±0.33 and 0.65±0.40 mm. Although the published results of a cadaveric study using SpineAssist for screw insertion revealed that 32 of the 36 placement (88.89%) were within ±1.5 mm of the planned positions with an overall deviation of 0.87±0.63 mm [10], and a retrospective 14-center study of SpineAssist reported that 98.3% of 646 implanted screws fell into class A or B of the Gertzbein and Robbins criteria with mean deviations of 1.2±1.49 and 1.1±1.15 mm on the axial and sagittal planes [21], there is no comparison between the accuracies of the RSSS and SpineAssist. Our experiment used only optimal conditions and was performed on in vitro cadaveric sheep spines. The spine specimens were rigidly fixed to the operating table, whereas the SpineAssist has been used on clinical patients for many years in a minimally invasive method. In real clinical use, the patient would be “flexible” and would fluctuate with breathing; furthermore, the markers may be less visible due to the surrounding anatomical structures. In addition, the reported limitations of the SpineAssist, such as the possibility of a shift in the entry point and the trajectory caused by surrounding soft tissues, may also affect the RSSS [22]. All these factors will affect the final accuracy.

We also deliberately designed the wrong trajectory, which penetrated into the canal, where we found that the robot detected the abnormality and automatically stopped the drilling. At present, the robot recognizes the state of drilling and prevents the potential penetration. However, the robot can hardly determine which cortical zone is being penetrated, which is still mainly judged by the navigation or X-ray information.

Several procedural evaluations indicated that the robot exhibited some degree of errors. To advance the robot, each error was analyzed as follows.

1. Errors related to the reconstruction of the 3D coordinates with C-arm images, which resulted from the presence of the electromagnetic fields in the vicinity of the C- arm [11]. This type of error remained a general problem whenever fluoroscopic imaging devices were used.
2. Errors within the registration procedures: A point-based method was used during the registration between the vertebrae and C-arm image. The robot also needed to be calibrated with the tracking system. All of these factors inevitably introduced errors. As shown in Table 1, the average errors of registration and calibration were 0.12±0.02 mm and 0.16±0.04 mm, respectively.
3. Errors with the tracking system: The limited precision of the optical tracking system, which was used in the registration and tracking processes, led to the introduction of this error. In this experiment, the error of tracking was 0.25 mm (supported by the NDI Company).
4. Errors resulting from the robot manipulator: Although the manipulator had a low chance of having manufacturing errors, there was still an inevitable slight difference in algorithms. The error of repeated positions of the robot is 0.05 mm.
5. Errors resulting from the slipping of the drill: As most entrance points for the pedicle screw were on the slope of the lateral aspect of the facet joint, the slope could become steep, giving rise to a lateral and caudal skidding of the drilling tip at the entrance point, which was also reported by other studies [12]. There is a need for further research and a more vigorous technique to eliminate this issue.

## Conclusions

The Robotic Spinal Surgery System (RSSS) might be helpful for improving the accuracy and safety of the pedicle screw insertion procedures. This system has satisfied the initial properties for a surgical robotic system. Moreover, this robot used the force signal and the navigation information to recognize the different drilling states and had the potential to reduce the implications. In future studies, we will attempt to improve the accuracy and reliability of the RSSS system, and extensive animal and in vitro experiments need to be conducted in the near future.
